# Acoustic measures of speech sound production in multilingual children: A scoping review protocol

**DOI:** 10.1371/journal.pone.0361605

**Published:** 2026-10-28

**Authors:** Shuzhen Wang, Karla N. Washington

**Affiliations:** 1 Department of Speech-Language Pathology, University of Toronto, Toronto, Ontario, Canada; 2 Rehabilitation Sciences Institute, University of Toronto, Toronto, Ontario, Canada; 3 The Institute for Education Research, University Health Network, Toronto, Ontario, Canada; 4 Children’s Voices Centre, Charles Sturt University, Bathurst, New South Wales, Australia; 5 University of Cincinnati, Cincinnati, Ohio, United States of America; The Education University of Hong Kong, HONG KONG

## Abstract

In most societies, using more than one language or language variety is the norm, yet the reference data against which children’s speech is judged derive largely from monolingual speakers. Systematic cross-linguistic variation is therefore liable to be read as atypical development, placing multilingual children at risk of misdiagnosis of speech sound disorder (SSD). This risk is most acute for closely related, understudied varieties, where difference and disorder overlap and community-specific data are scarce. Acoustic measures (e.g., voice onset time, vowel formants, segment durations) quantify fine-grained, continuous properties of speech production that categorical, transcription-based description obscures. No existing review, however, has mapped which acoustic measures have been applied to multilingual children, across which language varieties, or against which reference baselines their productions are interpreted. The objective of this review is to provide that map, showing where acoustic evidence is concentrated and where it is absent, to support clinical assessment and to identify where community-specific reference data are most needed. The review will follow updated Joanna Briggs Institute scoping review methodology and will be reported per the PRISMA Extension for Scoping Reviews. Searches will be conducted in MEDLINE (Ovid), Embase (Ovid), CINAHL (EBSCO), Web of Science Core Collection, and Linguistics and Language Behavior Abstracts, with grey literature sought through ProQuest Dissertations and Theses Global and conference proceedings; no date limits will be applied. Children from birth to 18 years are eligible regardless of clinical status, and a preschool subset (≤ 5;11) will be reported separately. After pilot calibration, two reviewers will independently screen and chart sources, resolving disagreements by discussion or a third reviewer. Charted data will be mapped through an acoustic-measure taxonomy crossed with language variety and reference baseline. This protocol has been registered on the Open Science Framework (OSF https://doi.org/10.17605/OSF.IO/R2EX5).

## Introduction

Worldwide, far more people speak two or more languages than speak only one [1]. Accordingly, children commonly grow up hearing and using more than one language, or more than one dialect or closely related variety of a single language, such as British, Canadian, Jamaican, and American English. Speech-language pathologists (SLPs) are therefore increasingly required to assess the speech of multilingual children and to make a consequential judgment: whether a child’s productions reflect community-typical variation or a speech sound disorder (SSD). This judgment is difficult because the same surface feature can be typical in one variety yet a marker of disorder in another, so that difference (i.e., features of the native language) and disorder cannot be distinguished from surface form alone. Misjudging them places multilingual children at risk of both over-identification, in which typical variation is treated as disorder, and under-identification, in which a genuine disorder is missed [2–4]. This risk is not hypothetical. In a landmark 1979 case, eleven African American English–speaking children were identified through a speech-language evaluation as having disordered language development, on the basis of features that were in fact characteristic of their community variety and were placed in remedial classes; the resulting trial exposed the need to revise speech-language diagnostic practice so that it accounts for children whose dialect differs from the mainstream variety [5]. This risk is also structural, not incidental: SLPs increasingly serve multilingual children whose language or variety they may not share [2]; indeed, only 8% of SLPs in the United States self-identify as multilingual service providers [6].

Two properties of multilingual speech development make this risk systematic. First, a multilingual child is not the sum of two monolinguals. In Grosjean’s [7,8] holistic view, a bilingual is an integrated whole in which the two languages coexist and interact. Cross-linguistic interaction of this kind, namely the transfer, acceleration, and delay effects delineated by Paradis and Genesee [9], is also observable in the fine phonetics of production. Mayr and Montanari [10] report an acoustic case study of two simultaneous trilingual sisters acquiring Italian, Spanish, and English. The mother’s Italian /k/ and the nanny’s Spanish /k/ both showed short-lag voice onset time (VOT), whereas the sisters produced English-like long-lag VOTs for Italian /k/ and short-lag VOTs for Spanish /k/. The authors interpreted the English-like Italian productions as evidence of cross-linguistic interaction and suggested that differences in input could help explain it: Italian input included native speakers and English-dominant peers, whereas Spanish input came from a single monolingual nanny. Multilingual speech sound production can therefore diverge from monolingual expectations in principled, theoretically motivated ways. Second, the reference standard against which that production is judged is itself typically monolingual. The developmental norms and standardized tools available for children’s speech have been developed predominantly for monolingual speakers [2]. These two properties compound: the more a variety’s typical development departs from a monolingual baseline, the more a monolingual norm will misclassify it.

This compounding is unevenly distributed across the world’s languages. Relatively mature reference data and assessment approaches exist for widely studied pairings such as Spanish–English, where converging-evidence frameworks, which combine multiple sources of assessment data to reach a diagnostic decision, have been developed to separate difference from disorder in dual language learners [11,12]. They are largely absent for closely related varieties such as Creole languages and dialects of American English, where difference and disorder overlap substantially and community-specific reference models are especially scarce [6,13]. In this understudied context, children’s performance has sometimes been interpreted against adult models drawn from the same linguistic community rather than against monolingual norms, for both speech sound production [14] and standardized language assessment [15]. On the bidialectal side, African American English (AAE) is marked by systematic durational features, such as distinctively long vowel durations in adult speakers [16]. Recent acoustic work has begun to examine such features in AAE-speaking children, though so far in small samples and without direct comparison across varieties [17]. The problem of interpreting multilingual children’s speech is thus, at root, a problem of what is measured and what it is measured against.

How the field measures speech sound production bears on both of these. Historically, multilingual children’s speech has been characterized through perceptual judgment and phonetic transcription. These methods are categorical and listener-dependent and are especially vulnerable to bias when the clinician does not share the child’s language or variety [2]; by reducing a continuous signal to discrete symbols, they are poorly suited to capturing the fine, gradient variation that distinguishes cross-linguistic and cross-dialectal production from disorder. Acoustic measures, such as VOT, vowel formants, segment and word durations, and spectral moments, quantify this gradient variation directly from the speech signal, offering a more objective and replicable window onto production. In the Jamaican Creole and Jamaican English (JamC/JamE) context, for example, León and colleagues have used acoustic approaches to characterize segmental production against community adult models [14] and to index developing speech-motor control through durational methods (VOT, vowel, and whole-word duration) that compare children with same-community adults [18]. Crucially, however, this greater objectivity does not by itself resolve the difference–disorder distinction. Extending these durational methods to the clinical question directly, León et al. [19] compared acoustic durations in the English productions of JamC/JamE preschoolers with and without suspected SSD and found few and inconsistent differences between the two groups. The authors concluded that durational measures alone may not be sufficient for SSD diagnosis in this variety. The lesson is not that acoustic measurement fails, but that its diagnostic value is conditional: which acoustic measures, applied to which language varieties, and interpreted against which reference baselines, actually separate difference from disorder remains unknown. Establishing that conditionality is precisely what a systematic map of the evidence base can provide.

Contemporary accounts of phonological development give this gradient information theoretical weight. The Articulatory-map (A-map) model treats children’s productions as shaped by the distributional properties of stored motor-acoustic traces of previous productions, so that variability carries information about the developing system rather than being noise [20]. Gradient learning models such as the Gradual Learning Algorithm likewise formalize how variable, noisy input is learned and how free variation is represented in a developing grammar [21]. On these accounts, accuracy and variability jointly reflect the state of a developing phonological system. Accuracy is typically indexed categorically through transcription, whereas variability is indexed continuously through acoustics. Acoustic measures of variability therefore add information when productions assigned to the same transcription category differ acoustically. This is relevant to closely related varieties, where systematic differences in timing or spectral properties may occur within a shared transcription category.

The population of interest spans childhood because the difference–disorder question concerns a developing system. The question arises while speech sound production is still being acquired, when typical variation and disorder are most difficult to distinguish. Within childhood, the preschool years carry particular clinical weight. SSD is typically first identified in this period; prevalence estimates for speech delay are highest in the early years, with a median of 7.8% at age 5 falling to 2.3% by age 7 [22]; and persistent SSD still affects an estimated 3.6% of children at 8 years, underscoring the stakes of early identification [23]. Identification in these years is also the least certain. In Law et al.’s [22] systematic review, no study of speech-delay prevalence in children younger than 5 met the inclusion criteria, which the authors suggested reflects the difficulty of establishing case status in the very early years. For multilingual children, judged against monolingual reference standards, this uncertainty is even greater.

Following the International Expert Panel on Multilingual Children’s Speech [4], the present protocol treats “multilingual” as an umbrella covering children who comprehend and/or produce two or more languages, regardless of the age at which those languages were learned. Here the term is broadened to encompass bidialectal children as well: children who comprehend or produce two dialects or closely related varieties of a single language, consistent with work on Creole languages and American Englishes [6]. To date, it remains unclear which acoustic measures have been applied to multilingual children, to which language varieties and pairings, and, critically, against which reference baselines those productions have been interpreted. Without this map, it is not possible to see where acoustic evidence is concentrated, where it is absent, or whether the community-specific reference models needed to interpret closely related varieties exist at all.

A scoping review is well suited to this question. It is designed to map the breadth of an emerging, heterogeneous evidence base and to clarify how a concept has been operationalized, rather than to answer a narrowly defined question or appraise effectiveness [24,25]. Preliminary scoping searches informed the review’s focus: broadening the search from acoustic to instrumental or articulatory techniques yielded only a handful of additional, off-target records, supporting the decision to concentrate on acoustic measures. To confirm that no equivalent review already existed, PubMed, JBI Evidence Synthesis, and Open Science Framework (OSF) Registries were searched (June 17, 2026); no published, in-progress, or registered review addressing acoustic measures of speech sound production in multilingual children was identified. The single related registration retrieved from OSF Registries concerned speech-language therapy intervention and service delivery for children with SSD, rather than the measurement of their speech sound production.

Published reviews likewise address adjacent but distinct questions. Hambly et al. [26] systematically reviewed 66 studies of bilingual speech sound production, but characterized that literature principally through accuracy-based, transcription-derived measures (e.g., percentage of consonants/vowels correct); acoustic measurement was not treated as a category of analysis. Bazzocchi and Washington [27] map current methods for evaluating SSD in multilingual preschoolers through the International Classification of Functioning, Disability and Health (ICF) [28], but examine assessment measures broadly rather than acoustic measurement of production. Crowe et al. [29] reviewed interventions for multilingual preschoolers with speech and language difficulties, rather than measurement. Wright Karem et al. [30] mapped measures of language abilities (via the ICF [28]), not speech sound production. None of these sources charts which acoustic measures have been applied to multilingual children, across which varieties, or against which reference baselines.

The objective of this review is therefore to map how acoustic measures have been used to study speech sound production in multilingual children (which measures, which language varieties and pairings, and which reference baselines) in order to characterize the current evidence base, to inform clinical assessment, and to identify where community-specific reference data remain to be developed. Although this map is motivated by the problem of distinguishing difference from disorder in SSD, its object is the acoustic measurement of speech sound production, which applies across clinical populations; the review therefore includes multilingual children irrespective of clinical status and charts that status rather than using it to restrict inclusion. For the same reason, the map spans the full course of childhood, from birth to 18 years, rather than being limited to the ages of greatest clinical concern. Within this birth–18 map, a preschool (≤ 5;11) subset is reported separately, so that the evidence most relevant to early identification can be seen distinctly.

## Materials and methods

### Review questions

Following the Population–Concept–Context (PCC) framework, this review addresses three questions. The population is multilingual children, including bidialectal children; the concept is the acoustic measurement of speech sound production, including the reference baselines against which children’s productions are interpreted; and the context is any setting, such as laboratory, clinical, educational, home, or community, in which such measurement is undertaken.

**RQ1. Which acoustic measures of speech sound production have been applied to multilingual children, and how is the evidence distributed?** Distribution is examined across language varieties and pairings (e.g., Spanish–English, Mandarin–English, Jamaican Creole–English), age groups (preschool, ≤ 5;11, vs. older children), and clinical status (typically developing, SSD, or other reported condition).

**RQ2. For the measures identified in RQ1, what aspect of production does each measure capture (for example, accuracy or variability), and against what reference baseline is it interpreted?** Candidate baselines include monolingual child norms, monolingual adult targets, same-community adult models, same-community multilingual or bidialectal child comparison groups, the child’s other language, published acoustic reference datasets, or no explicit baseline.

**RQ3. What methodological challenges and evidence gaps limit acoustic measurement with multilingual children, and where are these gaps most consequential?** Challenges of interest include thin coverage of particular varieties, absence of community-appropriate reference data, and inconsistent measurement methods across studies (e.g., differing acoustic measures, elicitation tasks, or reference baselines).

### Eligibility criteria

**Population**. Participants are children who understand and/or produce two or more languages, or two dialects of a single language (bidialectal children), irrespective of when those varieties were acquired. This review includes children of all childhood ages, from birth to 18 years; a preschool subset (up to 5;11, years;months) will additionally be reported separately. Children are eligible irrespective of clinical status: typically developing children, children with SSD, and children with other developmental or medical conditions (for example, developmental language disorder, autism, hearing loss, or preterm birth) are all included, provided the study reports acoustic measures of speech sound production as defined under Concept. Clinical status will be charted as a study characteristic and reported in the evidence map. Where a study includes both monolingual and multilingual participants, or both children and adults, it is eligible only where the multilingual children’s results are reported separately or can be extracted.

**Concept.** Studies must report at least one acoustic measure of speech sound production derived from the acoustic speech signal (for example, VOT, vowel formants, segment or word durations, or spectral moments), grouped as temporal/durational, spectral, and source/voicing measures (see the taxonomy in S2 Appendix). Studies examining only language outcomes (e.g., vocabulary, grammar, narrative) or relying solely on perceptual or transcription-based measures without acoustic analysis will be excluded. Studies in which acoustic features serve only to detect or classify a clinical condition (e.g., voice-based autism detection or preterm cry analysis), rather than to characterize speech sound production, will be excluded. Studies combining perception and production components are eligible provided they report at least one acoustic measure of speech sound production, yet only the production measures will be charted.

**Context.** No restrictions are placed on geographic region, country, or linguistic community; studies conducted in any part of the world are eligible, consistent with the global distribution of multilingualism and with the objective of identifying where acoustic evidence is concentrated and where it is absent (RQ1 and RQ3). Eligible studies may be conducted in any setting in which children’s speech is recorded and acoustically analyzed, including laboratory, clinical, educational, home, and community settings. No date limits are applied, and both majority-language and minority-language or understudied-variety contexts are eligible. Studies are not restricted by the societal status of the languages involved (for example, official, regional, immigrant, heritage, or Creole varieties). Eligible reports must be available in English, be accompanied by a reliable translation (a full-text translation provided by the publisher or obtained from the study authors), or be in a language that a member of the review team (the first author and two other trained lab members) can chart directly. Machine or AI translation will not be used for eligibility decisions or data charting. The review team can chart sources directly in English, Spanish, and Mandarin.

**Types of sources.** Experimental and quasi-experimental designs, observational designs (cohort, case-control, cross-sectional, case series, and individual case reports), and qualitative studies will be considered. Given the scoping objective, text and opinion papers and grey literature (theses, dissertations, conference proceedings) will also be considered where they report original acoustic data. Reviews, including systematic reviews, scoping reviews, narrative reviews, and meta-analyses, will not be included as primary sources for data charting; however, the reference lists of relevant reviews will be hand-searched to identify potentially eligible primary studies.

### Protocol, framework, and registration

This scoping review will be conducted in accordance with the updated Joanna Briggs Institute (JBI) scoping review methodology [25], which builds on the framework of Arksey and O’Malley [31] and the enhancements of Levac et al. [32]. Consistent with that methodology, this protocol defines the review’s objective, questions, and eligibility criteria, and sets out the planned approach to searching, source selection, data extraction, analysis, and presentation; these methods will be carried out in the review itself after protocol registration. The review will be reported in accordance with the PRISMA Extension for Scoping Reviews (PRISMA-ScR) [33], and a completed PRISMA-P checklist for this protocol is provided as S1 Checklist [34].

This protocol has been registered on the OSF prior to execution of the full search (OSF https://doi.org/10.17605/OSF.IO/R2EX5). Any substantive amendment to this protocol will be recorded as a dated OSF registration update and reported in the review. Because this protocol concerns an evidence synthesis of previously published sources, elements such as sample size calculation, participant recruitment, randomization, blinding, informed consent, safety considerations, and data saturation do not apply.

### Search strategy

A three-step search strategy will be used, consistent with JBI guidance. The search strategy was developed in collaboration with a research librarian: the concept blocks, overall strategy structure, and trial search queries were defined and refined for sensitivity across the target databases (S1 Appendix).

**Initial limited search.** An initial limited search of MEDLINE (Ovid) was undertaken on July 27, 2026 in Ovid MEDLINE(R) ALL to identify relevant articles on the topic. The words in the titles and abstracts of retrieved papers, and the index terms used to describe them, were analyzed to develop the full search strategy. The MEDLINE master strategy was validated against a set of known relevant seed studies (S1 Appendix, A2). The seed studies were verified against their source PDFs for bibliographic accuracy, population eligibility, and the presence of acoustic measures of speech sound production, and all seed studies were confirmed as MEDLINE/PubMed-indexed. The strategy retrieved 5,108 records and retrieved all ten seed studies (S1 Appendix, A2).

**Full search across all databases.** The full search strategy, incorporating all identified keywords and index terms, will be adapted to the syntax and indexing structure of each information source and executed in MEDLINE (Ovid), Embase (Ovid), CINAHL (EBSCO), Web of Science Core Collection, and Linguistics and Language Behavior Abstracts (LLBA; ProQuest). The full MEDLINE (Ovid) strategy is provided (S1 Appendix, A1.1), with database-adaptation outlines for the other sources (A1.2), to be finalized before execution. For grey literature, unpublished theses and dissertations will be sought through ProQuest Dissertations and Theses Global, and conference proceedings will also be searched; these grey-literature sources were determined in consultation with the research librarian (S1 Appendix, A1.3).

**Reference and citation searching.** The reference lists of all included sources, and of relevant existing reviews, will be screened for additional studies; forward citation searching of key included studies will also be conducted. Searches will be re-run prior to final analysis if the review period exceeds 12 months from the initial search date, and any newly identified eligible studies will be incorporated.

### Source selection

All retrieved sources will be imported into Covidence, and duplicate ones will be removed before screening begins. Screening will then proceed in two rounds, first by title and abstract and then by full text, using the screening forms provided in S3 Appendix (C1 and C2). In each round, two reviewers will assess every source independently against the eligibility criteria (see Eligibility criteria), working within Covidence so that all decisions are visible to the team and recording the reason for each exclusion.

Before full screening begins, the two reviewers will pilot the title/abstract criteria on a random 5% sample of sources to confirm that they are interpreting the criteria consistently. Full screening will proceed once agreement exceeds 80%; if agreement is lower, the team will discuss the discrepancies, clarify the criteria and screening guidance, and pilot a fresh sample before continuing. At the title/abstract stage, each source will be marked *Include*, *Exclude*, or *Maybe*, and any source whose eligibility is unclear will be carried forward to full text. Full texts will then be retrieved for all sources marked *Include* or *Maybe* and assessed independently by the two reviewers, who will record the primary reason for each exclusion for the PRISMA-ScR flow diagram (Fig 1). Where the reviewers disagree, they will first seek consensus through discussion, and any decision that cannot be resolved this way will be settled by a third reviewer. The number and languages of sources excluded because no translation or language speaker was available will be counted and reported in the review.

**Fig 1:**
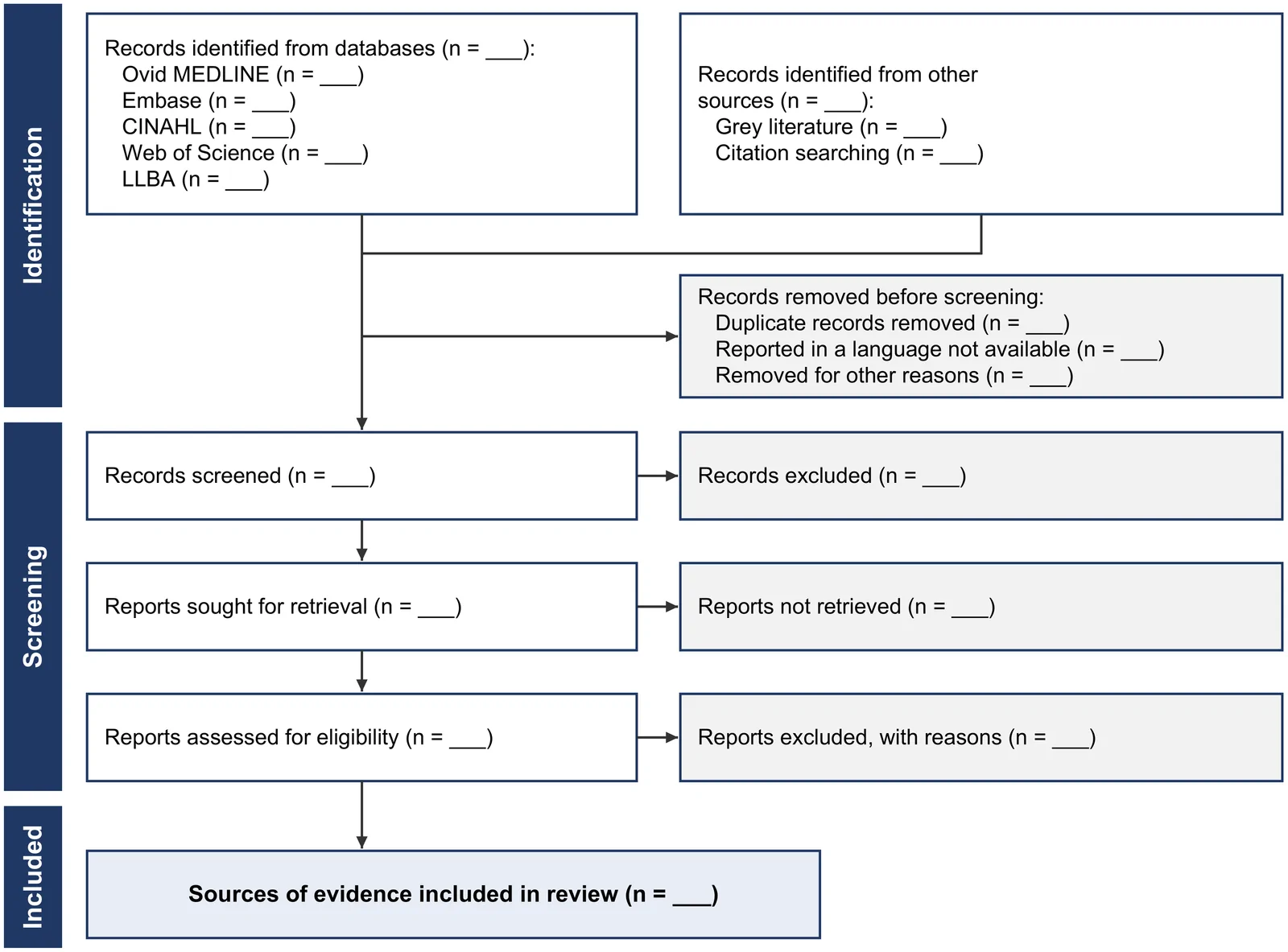
PRISMA-ScR flow diagram. The diagram shows the planned process for selecting sources of evidence. Counts (n) will be completed once the search and screening have been conducted.

### Data extraction (charting)

Data will be extracted (charted) from included sources independently by two reviewers using a data-charting form that will be piloted before use (S2 Appendix), developed by the reviewers and consistent with the JBI approach. The two reviewers will pilot the form on 5–10 included sources to calibrate charting before full charting begins. In addition to standard descriptive fields (authors, year, country/setting, study design, aims, participant numbers and ages, languages/varieties and pairing), the form is organized around an acoustic-measure taxonomy (temporal/durational, spectral, and source/voicing measures), crossed with what each measure is used to capture (accuracy vs. variability). The form additionally records the following: clinical status (typically developing, SSD, developmental language disorder [DLD], autism spectrum disorder [ASD], hearing loss or cochlear implant use, preterm birth, other, or mixed); the speech target and elicitation task (e.g., single-word naming, imitation, connected speech); the analysis software (e.g., Praat); and, critically, the reference baseline used to interpret children’s productions (baseline categories as specified in RQ2 and in S2 Appendix, B6). The draft charting form is expected to be modified as the review proceeds, and all modifications will be detailed in the final review. Modifications will be agreed by the review team, and any sources charted before a modification will be re-charted against the final form, so that all included sources are charted on identical fields. Disagreements will be resolved through discussion or, if needed, by a third reviewer. Where required data are missing or unclear, study authors will be contacted. Where authors do not respond within four weeks, the source will be charted on available information, and missing fields will be recorded as not reported.

Charting data will be stored in Covidence or a spreadsheet shared between the authors, with institutional backup. All data and materials underlying the findings of the completed scoping review will be made publicly available as Supporting Information when the final review article is published. These will include the final search strings for all databases searched, search dates and record counts, screening decisions, reasons for full-text exclusion, and the list of included studies. We will also provide the completed charting dataset.

### Data analysis and presentation

Consistent with scoping-review methodology, no critical appraisal (risk of bias) or meta-analysis will be undertaken, because the aim of a scoping review is to map the breadth of an evidence base rather than to appraise or synthesize effectiveness [24,25]. Extracted data will be analyzed descriptively and presented in two complementary forms. The first is a numerical summary of the amount, type, and distribution of included studies, tabulated by year, country, language variety and pairing, clinical status, age band, acoustic-measure category, and reference baseline, and presented in tables and charts. The second is a narrative map comprising (i) a language-variety-by-measure matrix showing which acoustic measures have been applied to which varieties and pairings (RQ1); (ii) a synthesis of what each measure captures (accuracy vs. variability) and the reference baselines against which productions are interpreted, cross-tabulated with measure type and clinical status (RQ2); and (iii) a sub-analysis of how studies address the difference–disorder distinction, with attention to closely related, understudied varieties (RQ3). The primary synthesis is organized around the contrast between typically developing children and children with SSD, which is central to distinguishing difference from disorder; other clinical groups are reported descriptively within the evidence map.

The rationale for the birth–18 scope is methodological rather than empirical. A scoping review aims to map the full extent of an evidence base; imposing a preschool ceiling at the search stage would prematurely narrow the map and risk excluding relevant school-age and adolescent acoustic studies. Because clinical concern often centers on the preschool years, the window in which SSD is typically first identified, a preschool subset (≤ 5;11) is reported separately within the full birth–18 dataset, so that clinically focused readers can locate the preschool evidence while the review retains the broader developmental picture. The full birth–18 map is the primary output of this review; the preschool subset is a pre-specified secondary stratum, registered in this protocol and reported within the full dataset rather than in place of it.

### Study status and timeline

At the time of submission, this study is at the protocol stage: the full search has not yet been executed, and no records have been screened or charted. The full search is expected to be executed in August 2026, with title/abstract and full-text screening completed by October 2026, charting and analysis by December 2026, and the completed review by February 2027.

### Stakeholder consultation

Consistent with the recommendation of Levac et al. [32] that consultation strengthens the methodological rigor of a scoping review, this review incorporates stakeholder consultation. The primary stakeholders are clinical SLPs serving multilingual communities; SLP perspectives are also represented within the review team. Following data synthesis and preliminary presentation of the findings, a targeted consultation with clinical SLPs will be conducted to gather expert feedback on the interpretation of the evidence map, its methodological limitations, and its clinical applicability. Insights from this consultation will be incorporated into the final interpretation and discussion of the completed review. The consultation plan will be finalized with the review team before the search is executed.

### Ethics and dissemination

Because this review synthesizes only previously published, publicly available sources, it does not require research-ethics board approval. Findings will be disseminated through a peer-reviewed publication, presentations at academic conferences, and knowledge-sharing activities with clinicians serving multilingual communities.

## Supporting information

S1 Appendix Search strategy (search strings by database; seed studies for search-strategy validation). (DOCX)

S2 Appendix Data-charting form. (DOCX)

S3 Appendix Screening forms (title/abstract and full-text). (DOCX)

S1 Checklist PRISMA-P checklist. (DOCX)
