# Supplementary material for: Acoustic measures of speech sound production in multilingual children: A scoping review protocol
Source: PLoS One. 2026 Oct 28;21(10):e0361605. doi: 10.1371/journal.pone.0361605 (PMC13716188; doi:10.1371/journal.pone.0361605)
Supplement: Supplementary file 1 [file pone.0361605.s001.docx]

**S1 Appendix. Search strategy**

**Overview**

The strategy combines three concept blocks with the Boolean operator AND: Block 1, multilingualism (population); Block 2, children (population age); and Block 3, speech-sound production and acoustic measurement (concept). Within each block, controlled-vocabulary (subject-heading) terms and free-text (title, abstract, and author-keyword) terms are combined with OR, following the two-place approach recommended by the research librarian (each concept searched as author title/abstract/keyword terms OR relevant subject headings). The final strategy is Block 1 AND Block 2 AND Block 3.

Block 3 combines speech-sound-production terms and acoustic-measure terms with OR (not AND) to maximize sensitivity, because these are two overlapping ways of describing the same concept. Whether a retrieved record actually reports an acoustic measure is decided at eligibility screening (see Eligibility criteria and the charting form in S2 Appendix), not by the search string. No language, date, or document-type limits are applied at the database level; language and study-type screening occur at selection.

Notation. * or $ = truncation; adjN (Ovid) / Nn (EBSCO) / NEAR/n (Web of Science) / N/n (ProQuest) = within-n-words adjacency; .ti,ab,kf. = title, abstract, and author-keyword fields (Ovid); .ti,ab,kw. = title, abstract, and keyword fields (Embase); .mp. = multi-field; / = subject heading (Ovid); MH = CINAHL heading; TS = topic (Web of Science); SU.EXACT = ProQuest subject heading.

**A1.1 Master strategy**

| **Ovid MEDLINE(R) ALL (1946 to July 24, 2026); searched July 27, 2026** | |
| --- | --- |
| **Block 1. Multilingualism (population)** | |
| 1 | Multilingualism/ |
| 2 | (bilingual* or multilingual* or trilingual*).ti,ab,kf. |
| 3 | (dual language or dual-language or two languages or second language or second-language or L2 or heritage language or minority language or immigrant language).ti,ab,kf. |
| 4 | (bidialect* or bi-dialect* or two dialects or dialect*).ti,ab,kf. |
| 5 | (English language learner* or ELL or emergent bilingual*).ti,ab,kf. |
| 6 | (((Spanish or Mandarin or Cantonese or Chinese or Korean or Japanese or Dutch or German or French or Arabic or Vietnamese or Tagalog or Punjabi or Hindi or Urdu) adj3 (speak* or speaker* or language* or bilingual* or monolingual* or accent* or dialect* or L1 or L2 or phonolog* or phonetic* or vowel* or consonant* or tone*)) or Creole or Jamaican or "African American English" or AAE).ti,ab,kf. |
| 7 | 1 or 2 or 3 or 4 or 5 or 6 |
| **Block 2. Children (population age)** | |
| 8 | exp Infant/ or exp Child/ or Child, Preschool/ or Adolescent/ |
| 9 | (child* or infant* or toddler* or preschool* or pre-school* or kindergarten* or "school age*" or "school-age*" or paediatric or pediatric or boy* or girl* or youth* or adolescen*).ti,ab,kf. |
| 10 | 8 or 9 |
| **Block 3. Speech-sound production and acoustic measurement (concept)** | |
| 11 | exp Speech/ or Speech Production Measurement/ or Phonetics/ or Speech Acoustics/ or Speech Sound Disorder/ or Articulation Disorders/ or Sound Spectrography/ or Acoustics/ |
| 12 | (speech sound* or speech production or speech-sound or articulat* or phonolog* or phonetic* or consonant* or vowel* or plosive* or stop* or fricative* or sibilant* or coda* or onset* or syllab* or segmental or suprasegmental*).ti,ab,kf. |
| 13 | (acoustic* or "voice onset time" or VOT or formant* or (F1 or F2 or F3) or duration* or (spectral adj2 (moment* or measure* or centroid or peak)) or "centre of gravity" or "center of gravity" or "fundamental frequency" or f0 or pitch or intensity or "cepstral" or praat or prosod* or intonation or "speech rhythm" or nasalance or "lexical tone*" or "tone production" or "tone language*" or "tonal language*" or tonal).ti,ab,kf. |
| 14 | 11 or 12 or 13 |
| **Combination** | |
| 15 | 10 and 7 and 14 |

**A1.2 Database adaptations**

**CINAHL (EBSCO). Replace Ovid headings with CINAHL headings and use TI/AB/TX fields.**

| **CINAHL (EBSCO)** | |
| --- | --- |
| S1 | (MH "Multilingualism") OR (MH "Bilingualism") |
| S2 | TI (bilingual* or multilingual* or trilingual* or bidialect* or "dual language" or "second language" or "heritage language" or "English language learner*") OR AB (same) |
| S3 | TI ((Spanish or Mandarin or Cantonese or Chinese or Korean or Japanese or Dutch or German or French or Arabic or Vietnamese or Tagalog or Punjabi or Hindi or Urdu) N3 (speak* or speaker* or language* or bilingual* or monolingual* or accent* or dialect* or phonolog* or phonetic* or vowel* or consonant* or tone*)) OR AB (same) |
| S4 | TI (Creole or Jamaican or "African American English" or AAE) OR AB (same) |
| S5 | S1 OR S2 OR S3 OR S4 |
| S6 | (MH "Child+") OR (MH "Infant+") OR (MH "Child, Preschool") OR (MH "Adolescence") |
| S7 | TI (child* or infant* or preschool* or "school age*" or pediatric or paediatric or adolescen*) OR AB (same) |
| S8 | S6 OR S7 |
| S9 | (MH "Speech+") OR (MH "Phonetics") OR (MH "Speech-Language Pathology") OR (MH "Articulation Disorders") OR (MH "Sound Spectrography") OR (MH "Acoustics") |
| S10 | TI (speech sound* or articulat* or phonolog* or consonant* or vowel* or fricative* or plosive* or syllab* or segmental or suprasegmental*) OR AB (same) |
| S11 | TI (acoustic* or "voice onset time" or VOT or formant* or duration* or spectral or "fundamental frequency" or f0 or pitch or praat or prosod* or intonation or nasalance or "lexical tone*" or "tone production" or tonal) OR AB (same) |
| S12 | S9 OR S10 OR S11 |
| S13 | S5 AND S8 AND S12 |

**Web of Science Core Collection (Topic field, TS).**

| **Web of Science Core Collection** | |
| --- | --- |
| #1 | TS=(bilingual* OR multilingual* OR trilingual* OR bidialect* OR "dual language" OR "second language" OR "heritage language" OR "minority language" OR "English language learner*" OR "emergent bilingual*") |
| #2 | TS=((Spanish OR Mandarin OR Cantonese OR Chinese OR Korean OR Japanese OR Dutch OR German OR French OR Arabic OR Vietnamese OR Tagalog OR Punjabi OR Hindi OR Urdu) NEAR/3 (speak* OR speaker* OR language* OR bilingual* OR monolingual* OR accent* OR dialect* OR phonolog* OR phonetic* OR vowel* OR consonant* OR tone*)) |
| #3 | TS=(Creole OR Jamaican OR "African American English" OR AAE) |
| #4 | #1 OR #2 OR #3 |
| #5 | TS=(child* OR infant* OR toddler* OR preschool* OR "school age*" OR pediatric OR paediatric OR adolescen*) |
| #6 | TS=(speech sound* OR articulat* OR phonolog* OR phonetic* OR consonant* OR vowel* OR fricative* OR plosive* OR syllab* OR segmental OR suprasegmental*) |
| #7 | TS=(acoustic* OR "voice onset time" OR VOT OR formant* OR duration* OR spectral OR "fundamental frequency" OR f0 OR pitch OR praat OR prosod* OR intonation OR nasalance OR "lexical tone*" OR "tone production" OR tonal) |
| #8 | #6 OR #7 |
| #9 | #4 AND #5 AND #8 |

**Embase (Ovid). Use Emtree headings; free-text blocks mirror MEDLINE, using .ti,ab,kw.**

| **Embase (Ovid)** | |
| --- | --- |
| 1 | 'multilingualism'/exp OR 'bilingualism'/exp |
| 2 | (bilingual* or multilingual* or trilingual*).ti,ab,kw. |
| 3 | (dual language or dual-language or two languages or second language or second-language or L2 or heritage language or minority language or immigrant language).ti,ab,kw. |
| 4 | (bidialect* or bi-dialect* or two dialects or dialect*).ti,ab,kw. |
| 5 | (English language learner* or ELL or emergent bilingual*).ti,ab,kw. |
| 6 | (((Spanish or Mandarin or Cantonese or Chinese or Korean or Japanese or Dutch or German or French or Arabic or Vietnamese or Tagalog or Punjabi or Hindi or Urdu) adj3 (speak* or speaker* or language* or bilingual* or monolingual* or accent* or dialect* or L1 or L2 or phonolog* or phonetic* or vowel* or consonant* or tone*)) or Creole or Jamaican or "African American English" or AAE).ti,ab,kw. |
| 7 | 1 or 2 or 3 or 4 or 5 or 6 |
| 8 | 'child'/exp OR 'preschool child'/exp OR 'infant'/exp OR 'adolescent'/exp |
| 9 | (child* or infant* or toddler* or preschool* or pre-school* or kindergarten* or "school age*" or paediatric or pediatric or adolescen*).ti,ab,kw. |
| 10 | 8 or 9 |
| 11 | 'speech'/exp OR 'speech analysis'/exp OR 'phonetics'/exp OR 'speech disorder'/exp OR 'articulation'/exp OR 'sound spectrography'/exp OR 'acoustics'/exp |
| 12 | (speech sound* or speech production or articulat* or phonolog* or phonetic* or consonant* or vowel* or plosive* or fricative* or sibilant* or syllab* or segmental or suprasegmental*).ti,ab,kw. |
| 13 | (acoustic* or "voice onset time" or VOT or formant* or duration* or (spectral adj2 (moment* or measure* or centroid or peak)) or "fundamental frequency" or f0 or pitch or intensity or praat or prosod* or intonation or nasalance or "lexical tone*" or "tone production" or tonal).ti,ab,kw. |
| 14 | 11 or 12 or 13 |
| 15 | 7 and 10 and 14 |

**LLBA (Linguistics and Language Behavior Abstracts, ProQuest). ProQuest field codes; LLBA indexes fine-grained phonetic terms, so the acoustic block is especially productive.**

| **LLBA (ProQuest)** | |
| --- | --- |
| 1 | SU.EXACT("Bilingualism") OR ti(bilingual* OR multilingual* OR trilingual* OR bidialect* OR "dual language" OR "heritage language") OR ab(bilingual* OR multilingual* OR trilingual* OR bidialect* OR "dual language" OR "heritage language") |
| 2 | ti((Spanish OR Mandarin OR Cantonese OR Chinese OR Korean OR Japanese OR Dutch OR German OR French OR Arabic OR Vietnamese OR Tagalog OR Punjabi OR Hindi OR Urdu) N/3 (speak* OR speaker* OR language* OR bilingual* OR monolingual* OR accent* OR dialect* OR phonolog* OR phonetic* OR vowel* OR consonant* OR tone*)) OR ab(same) |
| 3 | ti(Creole OR Jamaican OR "African American English" OR AAE) OR ab(same) |
| 4 | 1 OR 2 OR 3 |
| 5 | ti(child* OR infant* OR preschool* OR "school age*" OR adolescen*) OR ab(child* OR infant* OR preschool* OR "school age*" OR adolescen* OR acquisition) |
| 6 | ab(speech sound* OR articulat* OR phonolog* OR phonetic* OR consonant* OR vowel* OR fricative* OR syllab* OR segmental OR suprasegmental*) |
| 7 | ab(acoustic* OR "voice onset time" OR VOT OR formant* OR duration* OR spectral OR "spectral moment*" OR "fundamental frequency" OR f0 OR pitch OR prosod* OR intonation OR nasalance OR "lexical tone*" OR tonal) |
| 8 | 6 OR 7 |
| 9 | 4 AND 5 AND 8 |

**A1.3 Grey literature**

Grey-literature sources were determined in consultation with the research librarian: (1) ProQuest Dissertations and Theses Global, searched using the LLBA-style strategy above, and (2) conference proceedings. The specific conference-proceedings sources and the full search string for each will be documented in full when the search is executed.

**A2. Seed studies for search-strategy validation**

The seed set was selected to span acoustic-measure categories, language pairings, and child multilingual profiles. Seed studies were verified against the downloaded source PDFs as eligible for the review. All seed studies were confirmed as MEDLINE/PubMed-indexed: a search on the ten PubMed identifiers in Ovid MEDLINE(R) ALL on July 27, 2026 returned all ten records. The seed set was used to validate the MEDLINE master strategy, which was developed without reference to the terminology of the seed studies and retrieved all ten of them (10/10) when executed on July 27, 2026.

| **#** | **Seed study** | **Language pairing / population** | **Acoustic speech-production measure(s)** |
| --- | --- | --- | --- |
| 1 | Bunta et al. (2016), Initial stop voicing in bilingual children with cochlear implants and their typically developing peers with normal hearing | Spanish–English bilingual children (normal hearing and cochlear implants) | VOT; prevoicing |
| 2 | Fabiano-Smith et al. (2015), Acquisition of the stop-spirant alternation in bilingual Mexican Spanish–English speaking children | Mexican Spanish–English bilingual children | Intensity ratios for stop–spirant productions |
| 3 | Kehoe & Philippart de Foy (2023), The development of alveolar and alveopalatal fricatives in French-speaking monolingual and bilingual children | French-speaking monolingual and bilingual children | Spectral moments; fricative duration |
| 4 | León et al. (2022), Linguistically informed acoustic and perceptual analysis of bilingual children’s speech productions | Jamaican Creole–English bilingual preschoolers | VOT; whole-word duration; vowel duration; prevoicing |
| 5 | León et al. (2023), Characterizing speech sound productions in bilingual speakers of Jamaican Creole and English | Jamaican Creole–English bilingual preschoolers | VOT; vowel duration; whole-word duration; vowel-to-word duration proportion |
| 6 | Mayr & Montanari (2015), Cross-linguistic interaction in trilingual phonological development | Italian–Spanish–English trilingual children | VOT; lead voicing; stop/spirant realization |
| 7 | Mira et al. (2025), Phonemic and pitch variability in bilingual preschoolers | Jamaican Creole–English bilingual preschoolers | VOT; VOT variability; mean f0; f0 variability; prevoicing |
| 8 | Ng et al. (2010), Voice pitch characteristics of Cantonese and English produced by Cantonese–English bilingual children | Cantonese–English bilingual children | Speaking f0; pitch sigma; min and max f0; f0 range |
| 9 | Yang (2021), Vowel development in young Mandarin–English bilingual children | Mandarin–English bilingual children (with monolingual comparison) | F1/F2 formants; formant trajectories |
| 10 | Yang et al. (2025), The effect of language background and grade level on fricative production of children learning Mandarin in a Chinese–English bilingual program | Children in a Chinese–English bilingual program | Center of gravity; F2 onset |
