# Supplementary material for: Acoustic measures of speech sound production in multilingual children: A scoping review protocol
Source: PLoS One. 2026 Oct 28;21(10):e0361605. doi: 10.1371/journal.pone.0361605 (PMC13716188; doi:10.1371/journal.pone.0361605)
Supplement: Supplementary file 2 [file pone.0361605.s002.docx]

**S2 Appendix. Data-charting form**

Data-charting form developed by the reviewers, consistent with the JBI approach. Two reviewers will chart independently, and the form will be piloted on 5–10 included sources before full charting.

**B1. Source and study**

| **Field** | **Response type / options** |
| --- | --- |
| Covidence ID | auto |
| First author, year | text |
| Country / setting of data collection | text |
| Study design | experimental / quasi-experimental / cohort / case-control / cross-sectional / case series / single case / qualitative / text-opinion |
| Study aim(s) | free text |

**B2. Participants**

| **Field** | **Response type / options** |
| --- | --- |
| N (multilingual children analyzed) | number |
| Age (range and mean/SD) | record in years;months; note if within preschool subset (≤5;11) |
| Clinical status | typically developing / SSD / developmental language disorder (DLD) / autism spectrum disorder (ASD) / hearing loss or cochlear implant use / preterm birth / other (specify) / mixed |
| Language(s) / variety(ies) spoken | text |
| Language pairing / grouping | e.g., Spanish–English; JamC/JE; trilingual |
| Bi/multilingual vs. bidialectal | bilingual/multilingual / bidialectal |

**B3. Speech task and measurement**

| **Field** | **Response type / options** |
| --- | --- |
| Speech target(s) | specific phones / features |
| Elicitation task | single-word naming / picture / imitation / sentence / connected speech / other |
| Analysis software | e.g., Praat |
| Acoustic measure(s) | record category and specific name in B4 |

**B4. Acoustic-measure taxonomy (analytic core)**

*Tick all that apply and record the specific measure name(s).*

| **Category** | **Example measures** | **Recorded?** |
| --- | --- | --- |
| Temporal / durational | VOT; segment, vowel, or whole-word duration; speech/articulation rate | ☐ |
| Spectral | vowel formants (F1–F3); formant dynamics; fricative spectral moments; center of gravity | ☐ |
| Source / voicing | f0 / pitch; intensity; voice quality (e.g., HNR); prevoicing | ☐ |
| Other (specify) |  | ☐ |

**B5. What the measure captures**

| **Field** | **Response type / options** |
| --- | --- |
| Construct captured | accuracy / variability / both |

**B6. Reference baseline (analytic core; RQ2)**

| **Field** | **Response type / options** |
| --- | --- |
| Reference baseline used to interpret children’s productions | select one or more: monolingual child norms / monolingual adult targets / same-community adult models / same-community multilingual or bidialectal child comparison / the child’s other language / published acoustic reference datasets / no explicit baseline / other (specify) |
| Baseline source (citation / dataset) | free text |

**B7. Difference–disorder handling (RQ3)**

| **Field** | **Response type / options** |
| --- | --- |
| Addresses typical variation, SSD, or the difference–disorder distinction? | yes / no + brief note |
| Stated methodological challenges / limitations | free text |
| Author-identified evidence gaps | free text |
