# Supplementary material for: Acoustic measures of speech sound production in multilingual children: A scoping review protocol
Source: PLoS One. 2026 Oct 28;21(10):e0361605. doi: 10.1371/journal.pone.0361605 (PMC13716188; doi:10.1371/journal.pone.0361605)
Supplement: Supplementary file 3 [file pone.0361605.s003.docx]

**S3 Appendix. Screening forms**

**C1. Title/abstract screening form**

Reviewer: __________________________	Date: ______________

Inclusion criteria (retain the record if all are met or unclear):

(1) Children, birth to 18 years.

(2) Multilingual or bidialectal children.

(3) Concerns speech-sound production.

(4) Reports, or appears likely to report, an acoustic measure of speech-sound production.

| **Paper ID** | **Title** | **Decision (Include / Exclude / Maybe)** | **Annotations (reason to exclude or for uncertainty)** |
| --- | --- | --- | --- |
|  |  |  |  |
|  |  |  |  |
|  |  |  |  |
|  |  |  |  |
|  |  |  |  |
|  |  |  |  |
|  |  |  |  |
|  |  |  |  |
|  |  |  |  |

*Records marked Include or Maybe advance to full-text screening.*

**C2. Full-text screening form**

Reviewer: __________________________	Date: ______________

Inclusion criteria (all must be met for inclusion):

(1) Children, birth to 18 years.

(2) Multilingual or bidialectal children; in mixed samples, the multilingual children’s results are reported separately or can be extracted.

(3) Reports at least one acoustic measure of speech-sound production, used to characterize production rather than solely to detect or classify a clinical condition (e.g., voice-based autism detection, preterm cry analysis); not solely perceptual or transcription-based, language-only, or perception-only.

(4) Available in English; accompanied by a reliable translation (a full-text translation provided by the publisher or obtained from the study authors); or in a language a review-team member can chart directly. Machine or AI translation is not accepted for eligibility or charting.

(5) Primary source reporting original acoustic data; reviews (systematic, scoping, narrative, or meta-analyses) are not charted as primary sources.

| **Paper ID** | **Children (birth–18)** | **Multilingual or bidialectal** | **Acoustic measure of production** | **English / translation available** | **Primary source (not a review)** | **Decision (Include / Exclude)** | **Primary reason to exclude** |
| --- | --- | --- | --- | --- | --- | --- | --- |
|  |  |  |  |  |  |  |  |
|  |  |  |  |  |  |  |  |
|  |  |  |  |  |  |  |  |
|  |  |  |  |  |  |  |  |
|  |  |  |  |  |  |  |  |
|  |  |  |  |  |  |  |  |
|  |  |  |  |  |  |  |  |
|  |  |  |  |  |  |  |  |
|  |  |  |  |  |  |  |  |

*For each excluded source, record the primary reason (not children; not multilingual or bidialectal; no separable multilingual data; no acoustic measure of production; acoustic measures used only for detection or classification; perceptual/transcription or language-only; no translation or language speaker available; wrong publication type) for the PRISMA-ScR flow diagram.*
