# Supplementary material for: Acoustic measures of speech sound production in multilingual children: A scoping review protocol
Source: PLoS One. 2026 Oct 28;21(10):e0361605. doi: 10.1371/journal.pone.0361605 (PMC13716188; doi:10.1371/journal.pone.0361605)
Supplement: Supplementary file 5 [file pone.0361605.s005.docx]

**Response to Reviewers**

Manuscript ID: PONE-D-26-37933

Acoustic measures of speech sound production in multilingual children: A scoping review protocol

Dear Dr. Lee,

Thank you for the opportunity to revise our manuscript. We appreciate the time you and the reviewers have devoted to reviewing our work, as well as the thoughtful and encouraging comments. They have helped us improve the clarity of our manuscript.

We have addressed the points raised by the reviewers. As requested, we have included our point-by-point responses in this letter, a revised manuscript with tracked changes, and a clean version of the revised manuscript. We have also updated the page numbers in the PRISMA-P checklist to match the clean version of the revised manuscript. We have also made minor editorial changes, including removing the hyphen in "speech-sound" throughout the manuscript and title. These changes do not affect the content of the protocol and are shown in the revised manuscript with tracked changes.

**Reviewer 1**

**1. Data availability**

***Comment:*** *The protocol was a pleasure to read. It makes a very strong case for running the scoping review, and is very thoughtfully prepared and written. I chose "no" for question 4 ("Have the authors described where all data underlying the findings will be made available when the study is complete?") because even though the protocol does state that data will be made available, it does not state explicitly where. Since this is the focus of the question and hence clearly a concern for PLOS One, I thought it best to give the authors the opportunity to clarify this in the paper (OSF? as supplementary material to the paper in PLOS One itself? if this info is not yet known, it can be explained quickly where the availability of the data is mentioned).*

**Response:** Thank you for your comments. This manuscript presents a scoping review protocol and does not report newly collected primary data. The review will extract and chart information from published journal articles and grey literature. We understand that your comment refers to the data and materials generated during the review, including search documentation, screening records, and the completed charting dataset. We have revised the data availability plan. The review data and materials will be publicly available as Supporting Information when the final review article is published. The revised text reads:

“Charting data will be stored in Covidence or a spreadsheet shared between the authors, with institutional backup. All data and materials underlying the findings of the completed scoping review will be made publicly available as Supporting Information when the final review article is published. These will include the final search strings for all databases searched, search dates and record counts, screening decisions, reasons for full-text exclusion, and the list of included studies. We will also provide the completed charting dataset.”

**2. Clarification of the trilingual sisters’ VOT patterns**

***Comment:*** *l. 69-73, “The sisters…systems”: I struggled somewhat to understand this passage, maybe due to the fact that “identically” can be taken as meaning “identically to the daughters” or “they realized Spanish and Italian (plosives?) identically”. The conclusion drawn from this and expressed in the following sentence also gave me pause, because it hinges on whether the daughters are showing the same VOT as the parents or not (depending on the meaning of “identically”), and in the second case I couldn’t understand how the resulting conclusion was drawn.*

**Response:** Thank you for pointing out this ambiguity. We have revised the passage in the Introduction (original lines 69–73) to clarify who produced each VOT pattern and how the findings support the authors’ interpretation. The mother’s Italian /k/ and the nanny’s Spanish /k/ both had short-lag VOTs. In contrast, the sisters produced long-lag VOTs for Italian /k/ and short-lag VOTs for Spanish /k/. We now explain that the authors interpreted the sisters’ English-like long-lag VOTs in Italian as evidence of cross-linguistic interaction. Differences in language input are presented as a possible explanation for this pattern. The revised passage reads:

“The mother’s Italian /k/ and the nanny’s Spanish /k/ both showed short-lag voice onset time (VOT), whereas the sisters produced English-like long-lag VOTs for Italian /k/ and short-lag VOTs for Spanish /k/. The authors interpreted the English-like Italian productions as evidence of cross-linguistic interaction and suggested that differences in input could help explain it: Italian input included native speakers and English-dominant peers, whereas Spanish input came from a single monolingual nanny.”

**3. Connection between acoustic variability and transcription categories**

***Comment:*** *l. 129-131, “That is…category”: The choice of connector (“That is”) creates the expectation that the sentence will be an elaboration on/clarification of the content of the previous one but I could not understand what aspect was being elaborated on. It’s almost like a sentence was deleted between the two.*

**Response:** Thank you for pointing out the unclear connection here. We have revised the two sentences to explain that productions assigned to the same transcription category can differ acoustically and why this is relevant to closely related language varieties. The revised text now reads:

“Acoustic measures of variability therefore add information when productions assigned to the same transcription category differ acoustically. This is relevant to closely related varieties, where systematic differences in timing or spectral properties may occur within a shared transcription category.”

**4. Supporting Information numbering**

***Comment:*** *l. 529: The Prisma-P checklist is listed as S1. I would have expected it to be S4 since it is the 4th supplementary element provided. Please disregard my comment if it stems from a PLOS One guideline.*

**Response:** Thank you for checking this. PLOS One’s naming convention allows supporting information files to be numbered separately by type. Our submission includes three appendices (S1 Appendix, S2 Appendix, and S3 Appendix) and one checklist. Although the PRISMA-P checklist is the fourth supporting information file, it is the first checklist and is therefore labelled “S1 Checklist.” We have retained this label.

**Reviewer 2**

***Comment:*** *I found this a very clear and comprehensive presentation of a scoping review protocol on the topic of acoustic measures of speech sound production in multi-lingual children. Of merit is the use of a standard protocol (J Briggs), a comprehensive set of databases proposed for searching; clear identification and rationale for the research questions and their relevance to the clinical and research community in clinical linguistics and therapy; excellent consideration of context of international multilingualism and its relevance to speech and language therapy practice globally; reach beyond relatively well-known pairings such as Standard American English/African American English; English/Spanish; Standard English(es)/Jamaica English and so on. I have no questions or additions to recommend.*

**Response:** We thank the reviewer for the positive feedback on the clarity and methods of our protocol and its relevance to research and clinical practice. We also appreciate the reviewer’s recognition of its focus on diverse language varieties and multilingual contexts.

Sincerely,

**Shuzhen Wang, PhD**

Postdoctoral Fellow

Paediatric Language, Learning & Speech (PedLLS) Outcomes Lab

Department of Speech-Language Pathology, University of Toronto

On behalf of the authors
