# Supplementary material for: Acoustic measures of speech sound production in multilingual children: A scoping review protocol
Source: PLoS One. 2026 Oct 28;21(10):e0361605. doi: 10.1371/journal.pone.0361605 (PMC13716188; doi:10.1371/journal.pone.0361605)
Supplement: Peer review history

# Peer review: Decision Letter 0

**DOI:** 10.1371/journal.pone.0361605.r001

**Authors:** Albert Lee

29 Sep 2026

PONE-D-26-37933Acoustic measures of speech-sound production in multilingual children: a scoping review protocolPLOS One

Dear Dr. Wang,

Thank you for submitting your manuscript to PLOS One. We have now received reviews from two experts in the field. Both reviewers praised the rigor, clarity, and importance of your protocol. Based on their feedback and my own reading, I am pleased to invite you to submit a **Minor Revision** . ============================

Thank you for your patience while we secured expert peer review for your protocol. To expedite the final publication of your work, please note that your revised manuscript will be evaluated directly by me as Academic Editor and will not be sent back out for external re-review.

Please address the minor clarification points raised by Reviewer 1 in your point-by-point response and manuscript revision.============================

Please submit your revised manuscript by Oct 14 2026 11:59PM. If you will need more time than this to complete your revisions, please reply to this message or contact the journal office at plosone@plos.org. When you're ready to submit your revision, log on to https://www.editorialmanager.com/pone/ and select the 'Submissions Needing Revision' folder to locate your manuscript file.

Please include the following items when submitting your revised manuscript:

- A letter that responds to each point raised by the academic editor and reviewer(s). You should upload this letter as a separate file labeled 'Response to Reviewers'.
- A marked-up copy of your manuscript that highlights changes made to the original version. You should upload this as a separate file labeled 'Revised Manuscript with Track Changes'.
- An unmarked version of your revised paper without tracked changes. You should upload this as a separate file labeled 'Manuscript'.

If you would like to make changes to your financial disclosure, please include your updated statement in your cover letter. Guidelines for resubmitting your figure files are available below the reviewer comments at the end of this letter.

If applicable, we recommend that you deposit your laboratory protocols in protocols.io to enhance the reproducibility of your results. Protocols.io assigns your protocol its own identifier (DOI) so that it can be cited independently in the future. For instructions see: https://journals.plos.org/plosone/s/submission-guidelines#loc-laboratory-protocols. Additionally, PLOS One offers an option for publishing peer-reviewed Lab Protocol articles, which describe protocols hosted on protocols.io. Read more information on sharing protocols at https://plos.org/protocols?utm_medium=editorial-email&utm_source=authorletters&utm_campaign=protocols.

As the corresponding author, your ORCID iD is verified in the submission system and will appear in the published article. PLOS supports the use of ORCID, and we encourage all coauthors to register for an ORCID iD and use it as well. Please encourage your coauthors to verify their ORCID iD within the submission system before final acceptance, as unverified ORCID iDs will not appear in the published article. *Only*  the individual author can complete the verification step; PLOS staff *cannot*  verify ORCID iDs on behalf of authors.

We look forward to receiving your revised manuscript.

Kind regards,

Albert Lee

Academic Editor

PLOS One

Journal Requirements:

When submitting your revision, we need you to address these additional requirements.

1. Please ensure that your manuscript meets PLOS ONE's style requirements, including those for file naming. The PLOS ONE style templates can be found at

https://journals.plos.org/plosone/s/file?id=wjVg/PLOSOne_formatting_sample_main_body.pdf and https://journals.plos.org/plosone/s/file?id=ba62/PLOSOne_formatting_sample_title_authors_affiliations.pdf

2. Please provide a complete Data Availability Statement in the submission form, ensuring you include all necessary access information or a reason for why you are unable to make your data freely accessible. If your research concerns only data provided within your submission, please write "All data are in the manuscript and/or supporting information files" as your Data Availability Statement.

3. If the reviewer comments include a recommendation to cite specific previously published works, please review and evaluate these publications to determine whether they are relevant and should be cited. There is no requirement to cite these works unless the editor has indicated otherwise.

4. Please review your reference list to ensure that it is complete and correct. If you have cited papers that have been retracted, please include the rationale for doing so in the manuscript text, or remove these references and replace them with relevant current references. Any changes to the reference list should be mentioned in the rebuttal letter that accompanies your revised manuscript. If you need to cite a retracted article, indicate the article’s retracted status in the References list and also include a citation and full reference for the retraction notice.

[Note: HTML markup is below. Please do not edit.]

Reviewers' comments:

Reviewer's Responses to Questions

**Comments to the Author**

1. Does the manuscript provide a valid rationale for the proposed study, with clearly identified and justified research questions?

The research question outlined is expected to address a valid academic problem or topic and contribute to the base of knowledge in the field.

Reviewer #1: Yes

Reviewer #2: Yes

**********

2. Is the protocol technically sound and planned in a manner that will lead to a meaningful outcome and allow testing the stated hypotheses?

The manuscript should describe the methods in sufficient detail to prevent undisclosed flexibility in the experimental procedure or analysis pipeline, including sufficient outcome-neutral conditions (e.g. necessary controls, absence of floor or ceiling effects) to test the proposed hypotheses and a statistical power analysis where applicable. As there may be aspects of the methodology and analysis which can only be refined once the work is undertaken, authors should outline potential assumptions and explicitly describe what aspects of the proposed analyses, if any, are exploratory.

Reviewer #1: Yes

Reviewer #2: Yes

**********

3. Is the methodology feasible and described in sufficient detail to allow the work to be replicable?

Descriptions of methods and materials in the protocol should be reported in sufficient detail for another researcher to reproduce all experiments and analyses. The protocol should describe the appropriate controls, sample size calculations, and replication needed to ensure that the data are robust and reproducible.

Reviewer #1: Yes

Reviewer #2: Yes

**********

4. Have the authors described where all data underlying the findings will be made available when the study is complete?

The PLOS Data policy requires authors to make all data underlying the findings described in their manuscript fully available without restriction, with rare exception, at the time of publication. The data should be provided as part of the manuscript or its supporting information, or deposited to a public repository. For example, in addition to summary statistics, the data points behind means, medians and variance measures should be available. If there are restrictions on publicly sharing data—e.g. participant privacy or use of data from a third party—those must be specified.

Reviewer #1: No

Reviewer #2: Yes

**********

5. Is the manuscript presented in an intelligible fashion and written in standard English?

PLOS ONE does not copyedit accepted manuscripts, so the language in submitted articles must be clear, correct, and unambiguous. Any typographical or grammatical errors should be corrected at revision, so please note any specific errors here.

Reviewer #1: Yes

Reviewer #2: Yes

**********

6. Review Comments to the Author

Please use the space provided to explain your answers to the questions above and, if applicable, provide comments about issues authors must address before this protocol can be accepted for publication. You may also include additional comments for the author, including concerns about research or publication ethics.

You may also provide optional suggestions and comments to authors that they might find helpful in planning their study.

(Please upload your review as an attachment if it exceeds 20,000 characters)

Reviewer #1: The protocol was a pleasure to read. It makes a very strong case for running the scoping review, and is very thoughtfully prepared and written. I chose "no" for question 4 ("Have the authors described where all data underlying the findings will be made available when the study is complete?") because even though the protocol does state that data will be made available, it does not state explicitly where. Since this is the focus of the question and hence clearly a concern for PLOS One, I thought it best to give the authors the opportunity to clarify this in the paper (OSF? as supplementary material to the paper in PLOS One itself? if this info is not yet known, it can be explained quickly where the availability of the data is mentioned).

I have a few minor requests for clarification.

l. 69-73, “The sisters…systems”: I struggled somewhat to understand this passage, maybe due to the fact that “identically” can be taken as meaning “identically to the daughters” or “they realized Spanish and Italian (plosives?) identically”. The conclusion drawn from this and expressed in the following sentence also gave me pause, because it hinges on whether the daughters are showing the same VOT as the parents or not (depending on the meaning of “identically”), and in the second case I couldn’t understand how the resulting conclusion was drawn.

l. 129-131, “That is…category”: The choice of connector (“That is”) creates the expectation that the sentence will be an elaboration on/clarification of the content of the previous one but I could not understand what aspect was being elaborated on. It’s almost like a sentence was deleted between the two.

l. 529: The Prisma-P checklist is listed as S1. I would have expected it to be S4 since it is the 4th supplementary element provided. Please disregard my comment if it stems from a PLOS One guideline.

Reviewer #2: I found this a very clear and comprehensive presentation of a scoping review protocol on the topic of acoustic measures of speech sound production in multi-lingual children. Of merit is the use of a standard protocol (J Briggs), a comprehensive set of databases proposed for searching; clear identification and rationale for the research questions and their relevance to the clinical and research community in clinical linguistics and therapy; excellent consideration of context of international multilingualism and its relevance to speech and language therapy practice globally; reach beyond relatively well-known pairings such as Standard American English/African American English; English/Spanish; Standard English(es)/Jamaica English and so on.

I have no questions or additions to recommend.

**********

7. PLOS authors have the option to publish the peer review history of their article (what does this mean?). If published, this will include your full peer review and any attached files.

If you choose “no”, your identity will remain anonymous but your review may still be made public.

**Do you want your identity to be public for this peer review?**  For information about this choice, including consent withdrawal, please see our Privacy Policy.

Reviewer #1: No

Reviewer #2: No

**********

[NOTE: If reviewer comments were submitted as an attachment file, they will be attached to this email and accessible via the submission site. Please log into your account, locate the manuscript record, and check for the action link "View Attachments". If this link does not appear, there are no attachment files.]

To ensure your figures meet our technical requirements, please review our figure guidelines: https://journals.plos.org/plosone/s/figures

You may also use PLOS’s free figure tool, NAAS, to help you prepare publication quality figures: https://journals.plos.org/plosone/s/figures#loc-tools-for-figure-preparation.

NAAS will assess whether your figures meet our technical requirements by comparing each figure against our figure specifications.

# Author comment: Author response to Decision Letter 1

**DOI:** 10.1371/journal.pone.0361605.r002

11 Oct 2026

Please see the attached file "Response to Reviewers" for our point-by-point responses.

Attachment Submitted filename: Response_to_Reviewers_Oct11.docx

# Editor report: Decision Letter 1

**DOI:** 10.1371/journal.pone.0361605.r003

**Authors:** Albert Lee

13 Oct 2026

Acoustic measures of speech sound production in multilingual children: A scoping review protocol

PONE-D-26-37933R1

Dear Dr. Wang,

We’re pleased to inform you that your manuscript has been judged scientifically suitable for publication and will be formally accepted for publication once it meets all outstanding technical requirements.

Within one week, you’ll receive an e-mail detailing the required amendments. When these have been addressed, you’ll receive a formal acceptance letter and your manuscript will be scheduled for publication.

An invoice will be generated when your article is formally accepted. Please note, if your institution has a publishing partnership with PLOS and your article meets the relevant criteria, all or part of your publication costs will be covered. Please make sure your user information is up-to-date by logging into Editorial Manager at Editorial Manager® and clicking the ‘Update My Information' link at the top of the page. For questions related to billing, please contact billing support.

If your institution or institutions have a press office, please notify them about your upcoming paper to help maximize its impact. If they’ll be preparing press materials, please inform our press team as soon as possible -- no later than 48 hours after receiving the formal acceptance. Your manuscript will remain under strict press embargo until 2 pm Eastern Time on the date of publication. For more information, please contact onepress@plos.org.

Kind regards,

Albert Lee

Academic Editor

PLOS One

Additional Editor Comments (optional):

Reviewers' comments:

# Editor report: Acceptance letter

**DOI:** 10.1371/journal.pone.0361605.r004

**Authors:** Albert Lee

PONE-D-26-37933R1

PLOS One

Dear Dr. Wang,

I'm pleased to inform you that your manuscript has been deemed suitable for publication in PLOS One. Congratulations! Your manuscript is now being handed over to our production team.

At this stage, our production department will prepare your paper for publication. This includes ensuring the following:

* All references, tables, and figures are properly cited

* All relevant supporting information is included in the manuscript submission,

* There are no issues that prevent the paper from being properly typeset

You will receive further instructions from the production team, including instructions on how to review your proof when it is ready. Please keep in mind that we are working through a large volume of accepted articles, so please give us a few days to review your paper and let you know the next and final steps.

Lastly, if your institution or institutions have a press office, please let them know about your upcoming paper now to help maximize its impact. If they'll be preparing press materials, please inform our press team within the next 48 hours. Your manuscript will remain under strict press embargo until 2 pm Eastern Time on the date of publication. For more information, please contact onepress@plos.org.

You will receive an invoice from PLOS for your publication fee after your manuscript has reached the completed accept phase. If you receive an email requesting payment before acceptance or for any other service, this may be a phishing scheme. Learn how to identify phishing emails and protect your accounts at https://explore.plos.org/phishing.

If we can help with anything else, please email us at customercare@plos.org.

Thank you for submitting your work to PLOS One and supporting open access.

Kind regards,

PLOS One Editorial Office Staff

on behalf of

Dr. Albert Lee

Academic Editor

PLOS One
